# Luteolin: A Phytochemical to Mitigate *S.* Typhimurium Flagellin-Induced Inflammation in a Chicken In Vitro Hepatic Model

**DOI:** 10.3390/ani13081410

**Published:** 2023-04-20

**Authors:** Patrik Tráj, Csilla Sebők, Máté Mackei, Ágnes Kemény, Orsolya Farkas, Ákos Kákonyi, László Kovács, Zsuzsanna Neogrády, Ákos Jerzsele, Gábor Mátis

**Affiliations:** 1 Division of Biochemistry, Department of Physiology and Biochemistry, University of Veterinary Medicine, István utca 2., H-1078 Budapest, Hungary; 2 National Laboratory of Infectious Animal Diseases, Antimicrobial Resistance, Veterinary Public Health and Food Chain Safety, University of Veterinary Medicine, István utca 2., H-1078 Budapest, Hungary; 3 Department of Pharmacology and Pharmacotherapy, Medical School, University of Pécs, Szigeti út 12., H-7624 Pécs, Hungary; 4 Department of Pharmacology and Toxicology, University of Veterinary Medicine, István utca 2., H-1078 Budapest, Hungary; 5 Department of Animal Hygiene, Herd Health and Mobile Clinic, University of Veterinary Medicine, István utca 2., H-1078 Budapest, Hungary

**Keywords:** antioxidants, flavonoid, phytochemical, immunity, interleukin, lipid peroxidation, *Salmonella*, paratyphoid, poultry

## Abstract

**Simple Summary:**

The inadequate use of antibiotics has resulted in the emergence of resistant microbes which imply a major threat in human and veterinary medicine. Therefore, natural alternatives improving the productivity of farm animals should be investigated to replace the extensive antibiotic application. The primary goal of the study was to prove the anti-inflammatory and antioxidant activity of luteolin (a common phytochemical of vegetables with a flavonoid structure) in a chicken hepatic cell culture. To investigate the effect of luteolin, a model was established which could recapitulate the *Salmonella enterica* serovar Typhimurium-induced hepatic inflammation of chickens. The inflammatory response was triggered with the elementary unit of the organ of bacterial motility, flagellin, and freshly isolated, primary hepatic cell cultures were applied containing both hepatocytes, the functional cells of the liver, and non-parenchymal, inflammatory cells. Luteolin at a concentration of 4 µg/mL did not alter the viability and the membrane integrity of the cells and therefore proved applicable to counteract flagellin. In combination with flagellin exposure, luteolin reduced the elevated IL-8 release of the cultured cells. Moreover, it reduced the concentration of IFN-α, H_2_O_2_ and malondialdehyde and restored the level of IL-10 and the ratio of IFN-γ/IL-10. In conclusion, luteolin had an anti-inflammatory and antioxidant effect in a chicken hepatic cell culture mimicking *Salmonella enterica*-associated inflammation.

**Abstract:**

The use of natural feed supplements is an alternative tool to diminish the damage caused by certain bacteria, improving animal health and productivity. The present research aimed to investigate the proinflammatory effect of flagellin released from the bacterial flagellum of *Salmonella enterica* serovar Typhimurium and to attenuate the induced inflammation with luteolin as a plant-derived flavonoid on a chicken primary hepatocyte–non-parenchymal cell co-culture. Cells were cultured in a medium supplemented with 250 ng/mL flagellin and 4 or 16 µg/mL luteolin for 24 h. Cellular metabolic activity, lactate dehydrogenase (LDH) activity, interleukin-6, 8, 10 (IL-6, IL-8, IL-10), interferon-α, γ (IFN-α, IFN-γ), hydrogen peroxide (H_2_O_2_) and malondialdehyde (MDA) concentrations were determined. Flagellin significantly increased the concentration of the proinflammatory cytokine IL-8 and the ratio of IFN-γ/IL-10, while it decreased the level of IL-10, indicating that the model served adequate to study inflammation in vitro. Luteolin treatment at 4 µg/mL did not prove to be cytotoxic, as reflected by metabolic activity and extracellular LDH activity, and significantly reduced the flagellin-triggered IL-8 release of the cultured cells. Further, it had a diminishing effect on the concentration of IFN-α, H_2_O_2_ and MDA and restored the level of IL-10 and the ratio of IFN-γ/IL-10 when applied in combination with flagellin. These results suggest that luteolin at lower concentrations may protect hepatic cells from an excessive inflammatory response and act as an antioxidant to attenuate oxidative damage.

## 1. Introduction

The liver receives much of its blood supply via the portal vein from the alimentary tract, containing bacterial products and dietary antigens, while the rest of its vascularization comes from the systemic circulation involving the hepatic artery [1]. Blood from each of these sources passes through liver sinusoids to get rid of invading pathogens and their byproducts with the help of endothelial cells and the greatest population of fixed macrophages of the body, Kupffer cells. These non-parenchymal cells also produce inflammatory cytokines to induce acute-phase protein secretion of hepatocytes or to regulate the local inflammatory response [2]. Materials that pass through this barrier may be further entrapped after being opsonized by hepatocyte-produced complement factors and soluble pathogen recognition receptors (PRR). The liver, therefore, orchestrates the innate immune response of the body via filtering out pathogens and producing soluble factors with systemic effect [1,2].

*Salmonella enterica* serovar Typhimurium (*S.* Typhimurium) and Enteritidis (*S.* Enteritidis) are the primary causes of foodborne human salmonellosis worldwide [3]. Contaminated poultry meat and eggs are common sources of the pathogen. The infection in chickens over 3 days of age persists for 8–9 weeks without clinical symptoms; therefore, surveillance and eradication of *Salmonella enterica* (*S. enterica*) serovars accountable for salmonellosis is a major objective of the poultry industry [4,5]. In other cases, following the invasion from the gastrointestinal tract, *S.* Typhimurium multiplies in the liver and spleen and might cause systemic infection and high mortality in chickens infected soon after hatching [6,7]. Emerging *S. enterica* serovars and unconventional organic farms still manifest a human threat [3,8,9,10].

Intriguingly, much of the gene expression profile observed in enteropathogenic *S. enterica*-induced inflammation on T84 human colonic adenocarcinoma cell line modeling human salmonellosis was attributed to flagellin, the protein monomer of the bacterial locomotory organelles, flagella [11]. Flagellin (in 50–100 ng/mL concentrations) exerted an increase in cytokine gene expression and caused degranulation and oxidative burst in a chicken heterophil granulocyte cell culture [12,13,14]. The presence of free bacterial flagellin in the host organism is hypothesized to be associated with the damage or the faulty assembly of the flagellum. Characteristically, the highly conserved hidden core region of this motor protein induced the activation of the non-specific immune system considerably via a Toll-like receptor 5 (TLR 5), a type of PRR [15]. Wild *S.* Enteritidis strains triggered more severe histopathological changes in the liver of chicken hatchlings and colonized the caecum and the spleen more avidly in the first few days post-infection than aberrant non-motile or non-flagellated mutants [16].

TLR5 activation by flagellin results in the induction of proinflammatory cascades. Upon ligand binding, the TLR dimerizes and undergoes conformational changes required to attach its adjacent intracellular domain (Toll/IL-1 receptor) to adaptor molecules. One such adaptor molecule is myeloid differentiation factor 88 (MyD88), which activates nuclear factor kappa B (NF-κB) [17,18]. NF-κB is a transcription factor that can selectively bind to the DNA to enhance the expression of cytokine genes [19]. One possible mechanism of the cell to alleviate the proinflammatory signal is to mask the nuclear localization sequence (NLS) of NF-κB with the inhibitor of κB (IκB) diverting the transport to the nucleus [20]. Further, certain plant-, fungi- or bacteria-derived bioactive compounds interact with TLR signaling via different mechanisms of actions; therefore, the change in inflammatory and stress response triggered by flagellin could arise from the modification of either step of the complex TLR-5 cascade [21].

Flavonoids as plant metabolites can influence mucosal and cellular immunity, modulate the endocrine response of the body and reduce oxidative damage caused by reactive compounds [22]. In addition, the literature suggests that in chickens, some members of this molecular group have been shown to improve hematological parameters and attenuate increased inflammatory responses [22,23]. In vivo and in vitro studies classify several mechanisms via which flavonoids could elicit an anti-inflammatory effect. Inhibition of prostanoid biosynthesis and cellular second messengers of the inflammatory signaling pathways (protein kinases, phosphodiesterases) and the suppression of NF-kB-mediated transcriptional activation of proinflammatory cytokine genes are of high importance [22,24]. Therefore, the use of flavonoids in both human and animal nutrition, including the foraging of broiler chicken, may be of great importance in the future to reduce unnecessary antibiotic use to prevent antimicrobial resistance [23,25]. Luteolin is a flavonoid found in most edible greens and vegetables, for instance, celery, rosemary, thyme, peppers, carrots, buckwheat and cabbage, but it was identified in the vast majority of Magnoliophyta families and lower phyla of plants as well [25]. As a widespread flavonoid, luteolin might be a candidate to alleviate the flagellin-evoked inflammatory response in *S. enterica*-infected chickens because, in mammalian cells, its efficacy to mitigate cellular inflammatory cascades such as the NF-κB, mitogen-activated protein kinase (MAPK) and signal transducer and activator of transcription 3 (STAT3) responsible for signal transduction during TLR and cytokine receptor signals has been reported in the literature [24]. The major goal of the present study was to provide a sufficient in vitro model of *S.* Typhimurium-triggered hepatic inflammation by applying a hepatocyte–non-parenchymal cell co-culture of chicken origin and to investigate the putative immunomodulatory and protective action of luteolin in restoring physiological hepatocellular inflammatory and redox homeostasis.

## 2. Materials and Methods

### 2.1. Cell isolation and Establishment of Cell Cultures

Cell isolation was performed from three-week-old male Ross-308 hybrid broiler chickens using a three-step in situ perfusion technique as previously described by Mackei et al. [26]. The animals were raised and fed following the guidelines of the breeder. The experiment was executed in conformity with institutional policies, approved by the Local Animal Welfare Committee of the University of Veterinary Medicine Budapest and by the Government Office of Zala County, Food Chain Safety, Plant Protection, and Soil Conservation Directorate, Zalaegerszeg, Hungary (number of permission: GK-419/2020; approval date: 11 May 2020). After CO_2_ narcosis, the chicken was slaughtered via decapitation and fixed in dorsal recumbency. After disinfection of the skin with ethanol, the body cavity was opened and the liver was perfused through a cannula inserted into the gastropancreaticoduodenal vein. The right atrium was incised and a glass cannula was inserted into the atrial cavity through the incision site, forming the drainage during perfusion.

All chemicals applied for cell isolation, establishment and treatment of cell cultures and for the measurements were obtained from Merck KGaA (Darmstadt, Germany), except where the source of the chemical was specifically indicated. The buffers used for cell isolation were incubated at 40 °C and pre-oxygenated with carbogen (95% O_2_ and 5% CO_2_, 5 min per 100 mL). The buffers flowed through the inflow branch at a rate of 30 mL/min. First, 150 mL of Hanks’ Balanced Salt Solution (HBSS) buffer containing ethylene glycol tetraacetic acid (EGTA) (0.5 M) was used to exsanguinate the liver and to bind Ca^2+^ and Mg^2+^ ions associated with the extracellular matrix. Subsequently, 150 mL of HBSS was used to ensure that EGTA was completely washed out of the tissues and did not interfere with the digestion process during the last step. Finally, the liver was washed with 100 mL of HBSS supplemented with 7 mM CaCl_2_, 7 mM MgCl_2_ (final concentrations) and 100 mg type IV collagenase (Nordmark, Uetersen, Germany).

After perfusion, the liver was removed, the Glisson’s capsule was disrupted and the freshly gained primary cell suspension was gently filtered through three layers of sterile gauze sheets, subsequently getting incubated in ice-cold HBSS supplemented with 50 mL of bovine serum albumin (BSA) for 50 min to prevent cell adhesion. The fractions containing hepatocytes and non-parenchymal cells were then separated by multistep centrifugation. The cell suspension was centrifuged three times for 3 min (100× *g*) in Williams’ Medium E (WM), which was supplemented with 0.22% NaHCO_3_, 50 mg/mL gentamicin, 2 mM glutamine, 4 µg/L dexamethasone, 20 IU/L insulin, 0.5 µg/mL amphotericin B and 5% fetal bovine serum (FBS). After each step, the supernatant containing non-parenchymal cells was collected separately and the sediment comprising hepatocytes was resuspended in WM with the same supplements in addition, as indicated above. After the third centrifugation, 20 mL of hepatocyte-rich purified cell suspension was obtained after resuspension of the pellet.

To separate the non-parenchymal cell fraction, the previously obtained supernatants were centrifuged at 350× *g* for 10 min. This step removed residual hepatocytes and red blood cells. The supernatant was then subjected to centrifugation at 800× *g* for 10 min. The fraction enriched in non-parenchymal cells was gained by resuspension of the sediment obtained. The proportion of viable cells in each fraction was then determined by a trypan blue exclusion test in Bürker’s chamber, revealing that the percentage of viable cells exceeded 90% of the total cell count. Both cell suspensions were diluted to a final cell concentration of 10^6^ cells/mL. In a prior study, freshly isolated hepatocyte- and non-parenchymal-cell-dominated fractions were characterized using flow cytometry and immunofluorescent detection of particular macrophage and hepatocyte markers to standardize the method [26].

**Figure 1:**
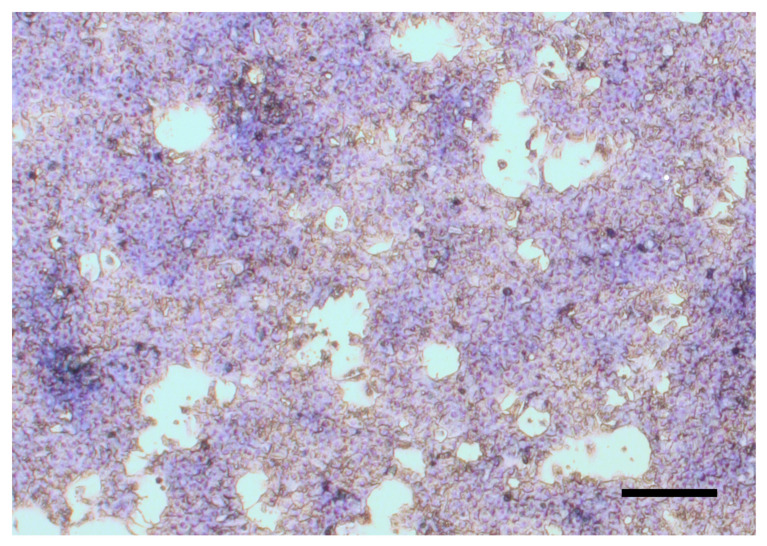
Giemsa staining of hepatocyte–non-parenchymal cell co-cultures after 24 h culturing (bar = 200 µm).

Cell fractions were mixed at a ratio of 6:1 (hepatocyte:non-parenchymal cells) and the final suspension was seeded in 24-well (400 µL/well) and 96-well (100 µL/well) culture dishes (Greiner Bio-One Hungary Kft, Mosonmagyaróvár, Hungary), coated with type I collagen (10 µg/cm^2^) according to the manufacturer’s instructions beforehand. Finally, cells were cultured in an incubator at 37 °C in a humid environment with 5% CO_2_. After four hours, the medium was replaced, and the confluency (approx. 90%) of the cultures was apparent after 24 h of incubation with Giemsa staining (Figure 1).

### 2.2. Treatment of Cell Cultures

After 24 h of incubation, cells were cultured in WM supplemented as detailed in Section 2.1 but without FBS and further supplemented with flagellin derived from *S.* Typhimurium at the concentrations of 0 (control) and 250 ng/mL, with 0, 4 or 16 µg/mL luteolin, or with the combination of flagellin (250 ng/mL) and luteolin (4 or 16 µg/mL, Cat. L9283, Merck KGaA) for 24 h. Flagellin and luteolin stock solutions were freshly prepared with pure WM.

The 400 µL/well culture medium of cells in 24-well plates was then collected, and they were lysed in 100 µL/well Mammalian Protein Extraction Reagent (M-PER) lysis buffer. Samples of cell lysate and culture medium were stored at −80 °C until the measurements.

### 2.3. Measurements

#### 2.3.1. Metabolic Activity

The metabolic activity of cells cultured in a 96-well plate was measured using the CCK-8 assay (Cell counting Kit-8, Dojindo Molecular Technologies, Rockville, MD, USA) according to the manufacturer’s instructions. The CCK-8 reagent contains Water Soluble Tetrazolium Salt (WST-8), which can be reduced by the NAD(P)H+H^+^ resulting from catabolic reactions. At the end of the 24 h treatment, cells were incubated for 2 h with 10 µL of CCK-8 reagent and 100 µL of FBS-free WM. The absorbance of the medium from each well was read at 450 nm using a Multiskan GO 3.2 instrument (Thermo Fisher Scientific, Waltham, MA, USA).

#### 2.3.2. LDH Activity

The extracellular lactate dehydrogenase (LDH) activity of the medium serves as an indicator of cell membrane integrity, where increased extracellular LDH activity refers to enzyme leakage due to membrane damage. The enzyme activity was determined using a kinetic photometric assay (Diagnosticum Ltd., Budapest, Hungary), performed by mixing 10 µL of culture medium with 200 µL of the reagent (56 mM phosphate buffer, pH 7.5; 1.6 mM pyruvate and 240 µM NADH+H^+^). The LDH activity was calculated by measuring absorbance at 340 nm in a Multiskan GO 3.2 instrument (Thermo Fisher Scientific, Waltham, MA, USA) at six time points with 1 min between each measurement and considering the mean of the differences between consecutive time points.

#### 2.3.3. IFN-α, IFN-γ, IL-10 Concentration

The Milliplex Chicken Cytokine/Chemokine Panel (Cat.Nr.: GCYT1-16 K, Merck KGaA, Darmstadt, Germany) was used to measure the concentration of IFN-α, IFN-γ and IL-10 protein in the medium according to the manufacturer’s instructions. All samples were thawed and analyzed in blind duplicates. A 96-well plate was filled with 25 µL of each sample, standard, control and reaction buffer. A further 25 µL of differently colored bead sets coated with primary antibodies were added to each well. Following the washing and overnight incubation steps, a biotinylated detection antibody mixture and phycoerythrin-conjugated streptavidin were applied to the plate. The beads were resuspended on the plate shaker for an additional five minutes following the addition of 150 µL of drive fluid into the wells. The fluorescence was measured by the Luminex MAGPIX^®^ instrument (Luminex Corporation, Austin, TX, USA). Data acquisition was performed using Luminex xPonent 4.2 software. Milliplex Analyst 5.1 software (Merck Millipore, Darmstadt, Germany) was used to generate five-parameter logistic regression curves as standard curves for each analyte using median values of fluorescence intensity of the beads.

#### 2.3.4. IL-6 and IL-8 Concentration

The concentrations of interleukin-6 (IL-6) and interleukin-8 (IL-8, syn. CXCLi2) were determined by a chicken-specific sandwich ELISA kit (MyBioSource, San Diego, CA, USA) according to the instructions of the manufacturer, and the absorbance was finally measured at 450 nm with a Multiskan GO 3.2 reader. The curve was fitted and concentrations were calculated with the free arigo GainData^®^ platform.

#### 2.3.5. H_2_O_2_ Level

The measurement of extracellular H_2_O_2_ level from the culture medium samples was performed using the Amplex Red assay (Thermo Fisher Scientific, Waltham, MA, USA). After incubation of 50 µL of Amplex Red (100 µM), HRP (0.2 U/mL) and 50 µL of culture medium at 21 °C for 30 min, the fluorescence of the samples was measured at 531 nm with an excitation set at 590 nm using a Victor X2 2030 fluorimeter (Perkin Elmer, Waltham, MA, USA).

#### 2.3.6. Malondialdehyde Concentration

The concentration of intracellular malondialdehyde (MDA) was determined from cell lysates. This product is formed during lipid peroxidation and can be measured using a lipid peroxidation (MDA) assay kit detecting thiobarbituric acid reactive substances. To 100 µL of the lysate samples, 300 µL of thiobarbituric acid was added, and the mixture was then incubated at 95 °C for 1 h according to the manufacturer’s instructions. This was followed by a 10-min cooling on ice, after which the absorbance of the sample was measured at 532 nm using a Multiskan GO 3.2 reader. The curve was fitted and concentrations were calculated with the free arigo GainData^®^ platform.

### 2.4. Statistical Analysis

Each measurement was performed with *n* = 6 replicates (well) per treatment group. Statistical analysis was carried out using R core Team software version 4.0.4. Results were plotted as the mean and standard deviation (SD) on bar graphs using GraphPad Prism (GraphPad Software Inc., San Diego, CA, USA). The measured concentrations of MDA, IL-6, IL-8, IFN-α, IFN-γ and IL-10 were standardized to the total protein concentration of the appropriate cell lysate as assessed with the BCA Protein Assay Kit (Thermo Fisher Scientific, Waltham, MA, USA). The Wilcoxon signed-rank test was applied to evaluate the significance of the differences between the absolute control (without flagellin and luteolin) and the single treatment (flagellin or luteolin) groups or between the flagellin-exposed and the combined-treatment (flagellin + luteolin) groups. The difference was considered significant if the *p*-value was 0.05 or less.

## 3. Results

### 3.1. Metabolic Activity and LDH Activity

**Figure 2:**
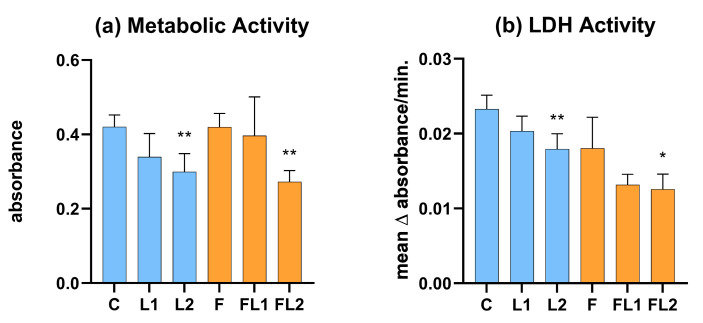
Metabolic activity measured by CCK-8 assay (**a**) and extracellular lactate dehydrogenase activity measured by an enzyme kinetic photometric assay (**b**). C = control, L1 = luteolin (4 µg/mL), L2 = luteolin (16 µg/mL), F = flagellin (250 ng/mL), FL1 = flagellin (250 ng/mL) and luteolin (4 µg/mL), FL2 = flagellin (250 ng/mL) and luteolin (16 µg/mL). Mean (*n* = 6/group) ± SD, * *p* < 0.05, ** *p* < 0.01. Group L1, L2 and F were compared to C, while group FL1 and FL2 were compared to group F.

The higher applied dose of luteolin (L2, 16 µg/mL) significantly (*p* = 0.002) reduced the metabolic activity of the cells (Figure 2a). This effect of the high luteolin concentration was also observed in combination with flagellin (FL2, *p* = 0.004). However, flagellin (F) or luteolin (L1) at a concentration of 4 µg/mL—both alone and in combination (FL1)—did not significantly alter the metabolic activity of the cells. The activity of lactate dehydrogenase released into the medium in response to cell membrane damage was significantly reduced by luteolin at 16 µg/mL, both in combination with flagellin (FL2, *p* = 0.030) and alone (L2, *p* = 0.009) (Figure 2b). Since luteolin was found to be cytotoxic at a concentration of 16 µg/mL, indicated by the decrease of the metabolic activity and the lactate dehydrogenase activity, the L2 and FL2 groups were excluded from the subsequent measurements.

### 3.2. IFN-α, IFN-γ, IL-10 Concentration and IFN-γ/IL-10 Ratio

**Figure 3:**
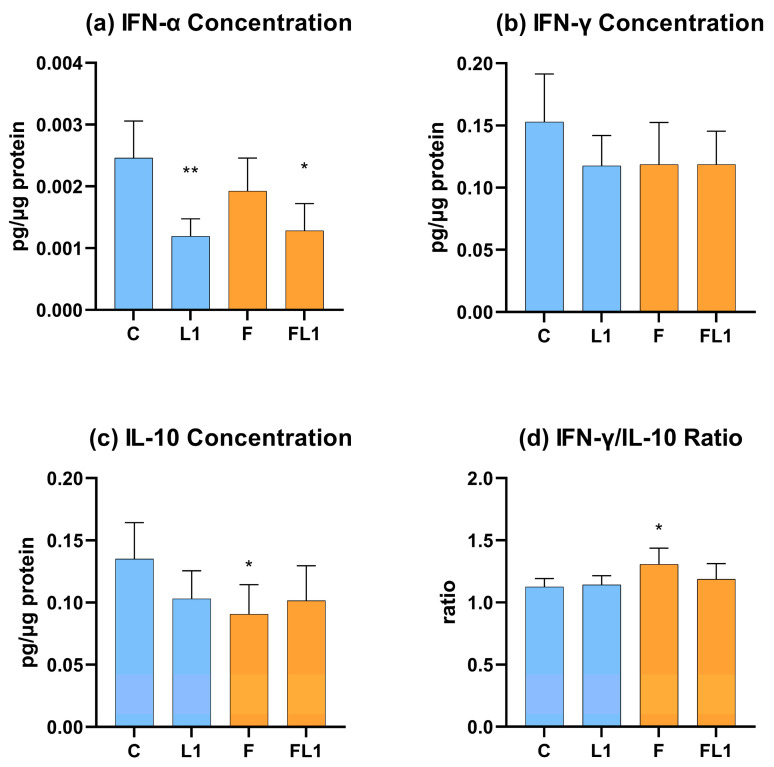
IFN-α (**a**), IFN-γ (**b**), IL-10 (**c**) concentration of the cell culture medium measured by chicken-specific Luminex MAGPIX Panel and the IFN-γ/ IL-10 ratio (**d**). C = control, L1 = luteolin (4 µg/mL), F = flagellin (250 ng/mL), FL1 = flagellin (250 ng/mL) and luteolin (4 µg/mL). Mean (*n* = 6/group) ± SD, * *p* < 0.05, ** *p* < 0.01. Group L1 and F were compared to C, while group FL1 was compared to group F.

The concentration of IFN-α was significantly decreased in the luteolin-treated groups (L1, FL1) compared to the control (C, *p* = 0.002) and the flagellin-exposed group (F, *p* = 0.041), respectively (Figure 3a). No significant change has been observed in the level of IFN-γ (Figure 3b). The concentration of IL-10 decreased in the case of sole flagellin exposure (F, *p* = 0.015); meanwhile, the level of the luteolin-cotreated (FL1) cells showed no significant difference in comparison with the control group (C) (Figure 3c). Further, the ratio of IFN-γ/IL-10 was elevated in the case of the flagellin group (F, *p* = 0.025) compared to the control (C) (Figure 3d).

### 3.3. IL-6 and IL-8 Concentration

**Figure 4:**
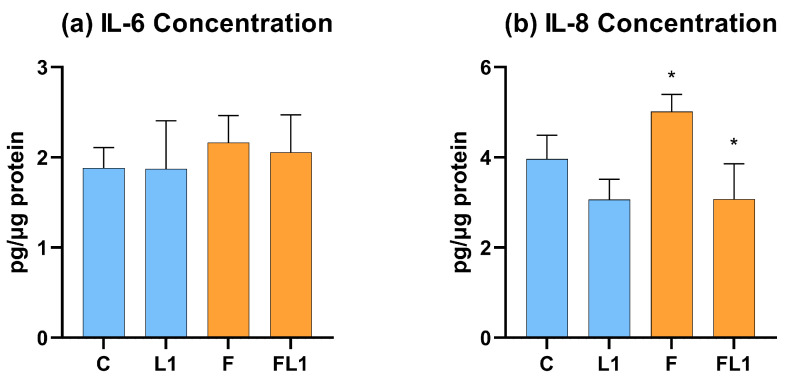
IL-6 (**a**) and IL-8 (**b**) concentration of the cell culture medium measured by chicken-specific ELISA. C = control, L1 = luteolin (4 µg/mL), F = flagellin (250 ng/mL), FL1 = flagellin (250 ng/mL) and luteolin (4 µg/mL). Mean (*n* = 6/group) ± SD, * *p* < 0.05. Group L1 and F were compared to C, while group FL1 was compared to group F.

No significant difference in IL-6 concentration was observed between any of the investigated treatment groups (Figure 4a). IL-8 concentration was significantly increased by flagellin treatment (F, *p* = 0.016) compared to the absolute control group (C). This increase was attenuated (*p* = 0.016) by the concomitantly applied luteolin (FL1) (Figure 4b).

### 3.4. H_2_O_2_ Level and Malondialdehyde Concentration

**Figure 5:**
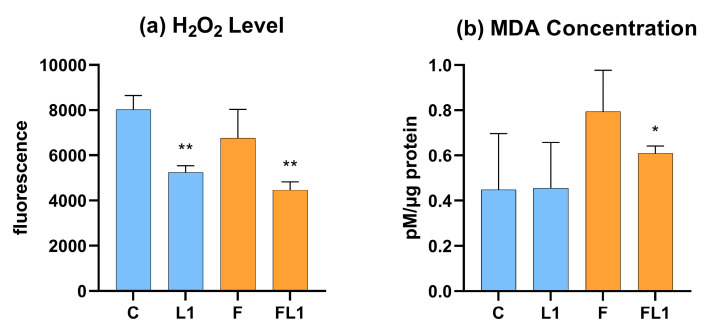
H_2_O_2_ level of the cell culture medium measured by Amplex Red fluorimetric assay (**a**) and MDA concentration of the cell lysate measured by a colorimetric assay (**b**). C = control, L1 = luteolin (4 µg/mL), F = flagellin (250 ng/mL), FL1 = flagellin (250 ng/mL) and luteolin (4 µg/mL). Mean (*n* = 6/group) ± SD, * *p* < 0.05, ** *p* < 0.01. Group L1 and F were compared to C, while group FL1 was compared to group F.

The decrease in extracellular H_2_O_2_ levels in response to luteolin (*p* = 0.002) was revealed by Amplex Red measurements both in the sole luteolin (L1) exposure group and in the combined flagellin–luteolin treatment group (FL1, *p* = 0.002) (Figure 5a). Similarly, as observed for H_2_O_2_ levels, MDA, a parameter indicative of membrane lipid peroxidation, showed a significant decrease (*p* = 0.016) in the luteolin and flagellin combined treatment group (FL1) compared to the level observed in the case of flagellin-exposed cells (F) (Figure 5b).

## 4. Discussion

With the emergence of microbes resistant to certain antibiotics, the WHO forecast warns us that antimicrobial resistance will imply a major threat: the number of deaths caused by resistant bacterial infections worldwide is predicted to increase 15-fold by 2050 [27,28]. Notwithstanding that the application of antimicrobials as growth promoters was banned in the EU in 2006, the still abundant and often prophylactic or metaphylactic overuse of these agents in the livestock sector can strongly increase the risk of resistance development [28,29]. In the poultry industry, there is growing evidence that the enhanced performance previously obtained with the subtherapeutic application of antibiotics is attainable with natural feed additives of plant origin [30,31,32]. The beneficial effect of these substances on animal health and productivity could be mediated by their anti-inflammatory, cytoprotective and antioxidant properties [22,33,34], which should be extensively investigated.

In the present study, a molecular pattern recognized by TLR5, flagellin of *S.* Typhimurium, was used to induce the hepatic inflammatory response. Following flagellin stimulation of chicken heterophilic granulocytes, Kogut et al. reported NFκ-B activation; an increase in IL-1β, IL-6 and IL-8 transcription; and described degranulation and free radical generation-inducing effects of the molecule [13,14]. Furthermore, recombinant *S.* Typhimurium flagellin stimulated IL-4, IL-6 and IL-12 [35], while *S.* Enteritidis infection in vitro elevated the IL-1β, IL-6 and IL-8 gene expression in chicken peripheral blood mononuclear cells [36]. These data accentuate the proinflammatory response of chicken inflammatory cell monocultures after flagellin exposure, albeit the liver and its resident cells are postulated primary targets of flagellated bacteria, and the TLR receptor toolkit of the organ might be crucial in the elimination of the bacterial burden from the portal circulation. A study on bioluminescent imaging of NF-κB-dependent luciferase expression in TLR5 agonist-treated mice unveiled that the liver is the major organ to respond to flagellin-like products in contrast to bacterial lipopolysaccharide (LPS); a surge of luminescence was detected uniquely in hepatocytes early after the TLR5 agonist administration [37].

The liver is one designated place of paratyphoid *S. enterica* multiplication in chickens; therefore, it could damage the organ in case of early infection of hatchlings [6,16]. Hence, the major aim of our work was the in vitro modeling of inflammation and oxidative stress induced by ciliated bacteria in primary hepatocyte–non-parenchymal cell co-cultures of chicken origin to recapitulate the response of the avian liver. Based on the previous studies, the applied primary co-culture can be considered a proper model of avian hepatic inflammation, mimicking moderate intrahepatic macrophage migration with a cell ratio of 6:1 (hepatocytes to non-parenchymal cells). In recent years, the authors examined the effect of specific pathogen-associated molecular patterns with the established and characterized primary co-culture [26]. Previous studies confirmed that flagellin at concentrations of 100 and 250 ng/mL did not induce cellular damage in terms of increased LDH activity or decreased metabolic activity of the cells. However, flagellin significantly increased the production of proinflammatory IL-6 in the 2D model at 250 ng/mL and in the 3D model at 100 ng/mL [38]. The present study reports a significant elevation of the expression of IL-8 protein in the 2D chicken primary culture of hepatocyte–non-parenchymal cells, which is in agreement with the data published on chicken heterophilic granulocytes and peripheral blood mononuclear cells.

The effect of luteolin on cell culture of chicken origin was investigated for the first time to the best of our knowledge; however, luteolin has already been applied on a large scale of mammalian cell lines. The protective effect of the molecule on cell viability and membrane integrity was confirmed on NRK-52E rat kidney cells when applied at as high as 50–200 μM concentration [39]. Wang et al. (2021) examined the cellular metabolic activity of mouse primary hepatocyte culture following LPS exposure. The authors have reported that in relatively low, 10 and 20 μM, concentrations, it has restored the viability of the cells [40]. Luteolin also served as a cytoprotective agent under LPS and ATP induced inflammatory cell death in 50 μM in human THP-1 monocyte cell line indicated by decreased LDH leakage and restored cell metabolic activity. This concentration proved to be the highest tolerable to the monocyte culture as 100 μM luteolin induced a significant decrease in cellular viability [41]. In a hepatic cell culture model of the present study, 16 μg/mL concentration, a dose similar to 50 μM (equal to 14.132 μg/mL) of luteolin, proved to be cytotoxic. In contrast, exposure to low concentrations of luteolin (5 μM, referring to 1.413 μg/mL) in RAW264.7 macrophages effectively modified the inflammatory response of cells without any effect on LDH-indicated cytotoxicity, in consonance with the 4 μg/mL dosage applied in the present study [42].

IFN-γ in chicken, similarly to mammalian species, is a key cytokine of the immune system as the primary activator of phagocytosis and a regulator of T helper cell function [43,44,45]. It is hypothesized to be a vital cytokine to terminate the spread of *S. enterica* as it contributes to the elimination and processing of intracellular pathogens in phagocytes [46]. Both human and murine model studies confirmed that high IFN-γ and low IL-10 levels were connected to the successful activation of the immune system and the elimination of enteric salmonellosis in the early phase of the infection [46,47,48]. The level of the cytokine is not proven to be associated with the pace of the clearance in chicken, although the escalation of the IFN-γ level in the spleen and cecal tonsils is observed to be vital to eliminate *S. enterica* from the gastrointestinal tract [49]. The present study, therefore, aimed to examine the changes in the level of IFN-γ and IL-10, two crucial cytokines in paratyphoid *S. enterica*-induced response, and their ratio consequently. The decrease observed in IL-10 concentration and the increase in IFN-γ/IL-10 ratio in case of flagellin exposure corroborate with a proinflammatory response similar to that observed in mammals and therefore validate the applied in vitro model [46,47,48]. Type I interferons, such as IFN-α are essential for efficient immunity, while their sustained synthesis inhibits macrophage and NK cell activity and leads to the production of ROS. Similarly, excessive IFN-γ production locally could lead to a harmful cytotoxic reaction; the elevation of this cytokine is a characteristic of the etiology of virus-induced tissue damage (e.g., infectious bursal disease, chicken infectious anemia) [50,51,52]. In the present study, luteolin effectively diminished the hepatic IFN-α release both in sole and combined application with flagellin, indicating the suggested anti-inflammatory role of the flavonoid in maintaining the physiological inflammatory homeostasis.

Luteolin exposure at 4 μg/mL decreased the IL-8 but not the IL-6 production of chicken hepatic co-cultures. Chicken IL-8 (syn. CXCLi2) attracts monocytes, macrophages and lymphocytes to the site of inflammation, but in higher concentrations, it also stimulates angiogenesis [53]. IL-8 production of human HT29 colon adenocarcinoma cells following the TNF-α challenge was diminished by luteolin [54]. In another study, luteolin reduced the accumulation of the proinflammatory cytokines IL-6 and TNF-α induced by LPS exposure in mouse primary hepatic cell cultures [40]. These results could be further supported by the observation of Wang et al. In their study, RAW264.7 macrophages were treated with LPS and IFN-γ to generate cultures of proinflammatory elongated M1 phenotype macrophages. Subsequently, luteolin treatment resulted in a concentration-dependent alteration in cell morphology towards the appearance of mainly anti-inflammatory rounded M2 macrophages and a significant decrease in gene expression of the cytokines IL-1β and IL-6 [55]. The present study demonstrates that luteolin could decrease the protein level of IL-8 in a chicken primary co-culture recapitulating the cell ratio of the avian liver. The mechanism of action in mammalian cells is presumed to be connected to inhibitors of NF-κB (IκBs), which prevent NF-κB from binding to the DNA and set forth the production of proinflammatory cytokines [24,42,54,56]. The blockage of the NF-κB signal might be a putative mechanism in chickens as well, as no remarkable difference between the TLR5 signal pathway in avian and mammalian cells has been described; the flagellin-induced alarm of the immune system is highly conserved [57].

Macrophages, monocytes, neutrophils, avian heterophils and eosinophilic granulocytes perform phagocytosis to remove pathogens from the body. The various reactive substances produced can also be released into the interstitial space during degranulation of the inflammatory cells, thus being able to not only damage pathogens but also oxidize the proteins, nucleic acids and lipid components of the cells themselves in the microenvironment of inflammation [58,59,60]. In birds, heterophilic granulocytes function as non-oxidative phagocytes; therefore, macrophages and monocytes are of key importance [61]. Excessive stress and disturbance of the redox signaling could lead to the overproduction of reactive species [62]. This oxidative stress could be compensated by nutritional antioxidants or by stimulating the production of antioxidant enzymes (e.g., superoxide dismutase, glutathione peroxidase, heme oxygenase) regulated by nuclear factor-erythroid 2 coupled factor 2 (Nrf2) [62,63]. The activation of the Nrf2 signaling pathway may be one of the potential mechanisms by which luteolin diminishes cellular oxidative stress [56]. Flavonoids are able to structure-specifically scavenge reactive oxygen species and reduce the rate of lipid peroxidation [64]. The results of the present study confirm that luteolin could alleviate lipid peroxidation and decrease the extracellular H_2_O_2_ level. In line with the observation of the authors, a decrease in the intracellular reactive oxygen species level was reported in cardiomyocytes and IPEC-J2 intestinal porcine enterocyte cultures after luteolin supplementation [56,65].

The present in vitro model study has provided some initial evidence concerning the anti-inflammatory and antioxidant role of luteolin in the chicken liver, suggesting that it might be a potential and safe candidate in poultry nutrition to ameliorate the deteriorative effects of enteric bacterial infections. However, it has to be stressed that further studies are required to assess the effects of this plant-derived metabolite even under in vivo conditions when applied as a feed additive.

## 5. Conclusions

In summary, the results of the present study confirmed that the applied primary hepatocyte–non-parenchymal cell co-culture could be a proper tool for modeling the avian hepatic inflammatory and stress response triggered by *S.* Typhimurium flagellin. The applied dose of flagellin (250 ng/mL) was not cytotoxic, but it could provoke inflammation as indicated by the increased cellular IL-8 and decreased IL-10 production and by the elevated IFN-γ/IL-10 ratio, which were effectively restored by luteolin at the dose of 4 µg/mL. Besides its anti-inflammatory action, luteolin was also capable of remarkably decreasing the extracellular H_2_O_2_ and MDA concentrations indicative of oxidative stress and lipid peroxidation. Based on these data, it can be suggested that luteolin might be a potential natural candidate to maintain the physiological inflammatory and redox homeostasis of the liver in chicken, possibly mitigating the destructive action of flagellin-associated inflammation caused by enteric bacterial infection. Hence, the administration of luteolin or some other flavonoids might be a promising tool to improve animal health and to reduce antibiotic application in poultry farming, which should be also addressed by further in vivo studies.

## Data Availability

All raw data supporting the results of the present study can be obtained from the corresponding author upon reasonable request.
